# Editorial: Developmental genes and molecular approaches: From embryo to human diseases

**DOI:** 10.3389/fnmol.2022.1089411

**Published:** 2022-11-23

**Authors:** Tania C. L. S. Spohr, Rolf Mentlein, John O. Mason

**Affiliations:** ^1^Centogene GmbH, Rostock, Germany; ^2^University of Kiel, Kiel, Germany; ^3^Centre for Discovery Brain Sciences, University of Edinburgh, Edinburgh, United Kingdom; ^4^Simons Initiative for the Developing Brain, University of Edinburgh, Edinburgh, United Kingdom

**Keywords:** signaling pathways, molecular biology, organogenesis, tumorigenesis, human diseases

As editors, we are very proud to present this Research Topic. It was really gratifying to review all the accepted reviews and research articles published in this Research Topic. In this editorial, we want first to thank all the reviewers who helped us with this task and to introduce our topic, and briefly summarize the contributing articles.

Our Research Topic focuses on developmental genes and their involvement in normal and disease development. Key processes essential for normal embryonic development include cellular proliferation, migration, and differentiation, all of which are regulated by crucial signaling pathways whose dysregulation often leads to developmental defects and severe pathologies.

Regarding unregulated signaling pathways in pathologies, Hu et al. reviewed the potential roles of genetic variants in thousand and one (TAO) kinases, which are major contributors to several neurodevelopmental disorders (NDDs). In this review the authors summarize the potential roles of the TAO kinases based on the development of neurodevelopmental disorders. The group focused on biochemical and structural analyses, by presenting the genetic data from clinical investigations. Furthermore, the group also discuss the mechanistic link between neuropathology, the mutations of TAO kinases, and behavioral impairment in NDDs.

Another gene family known to play important roles in the development of several pathologies is the *tweety* gene family. This family was very well-reviewed by Nalamalapu et al. in a review published in 2021, covering *tweety* gene family evolution, structure, expression during adulthood and development, cellular and biochemical functions, and their role in human disorders [including Alzheimer's disease, status epilepticus, amyotrophic lateral sclerosis (ALS), and Parkinson's disease].

The canonical Wnt/β-catenin pathway is an important regulator of cell fate during embryo development and adult neurogenesis. Therefore, the pharmacological manipulation of this pathway is widely used and of broad interest. Using stable human neural precursor cell (hNPC) lines derived from embryonic stem cells, Telias and Ben-Yosef investigated the effects of Wnt/β-catenin signaling on neuronal differentiation using the glycogen synthase kinase-3β inhibitor CHIR99021, to upregulate Wnt β-catenin signaling and the tankyrase-1/2 inhibitor XAV939 to downregulate it. The authors found that Wnt-agonism promoted induction of neural differentiation, while also reducing cell proliferation and survival. This effect was not synergistic with those of pro-neural growth factors during long-term neuronal differentiation. Conversely, antagonism of Wnt by XAV939 consistently prevented neuronal progression of hNPCs. The paper nicely illustrates how these two drugs can be used to manipulate cell fate and how self-renewing hNPCs can be used as reliable human *in vitro* drug-screening platforms.

Another research paper from Lim et al. in this Research Topic studied the role of serine-threonine kinase WNK3 (With No Lysine [K]), in GABAergic signaling during maturation of prenatal hippocampal neurons. Their study provides the first evidence that WNK3 plays a crucial role in maintaining the polarity of GABAergic signaling, neuron morphology, intrinsic excitability, and synaptic excitation in mature hippocampal neurons in either a KCC2-dependent or independent manner. The authors demonstrate that WNK3 deficiency induces EGABA hyperpolarization, indicating an increase in GABA inhibitory response in mature neurons, through the upregulation of KCC2 activity. In addition, WNK3 deficiency in mature neurons led to altered neuron morphology consistent with the shift of GABAergic inhibitory response upon upregulation of KCC2 activity. The group concluded that WNK3 possibly affects neuronal somatic and synaptic properties by modulating KCC2 activity, resulting in abnormal activity patterns that may underlie psychiatric and neurological disorders.

As the immune system also plays a crucial role in several disorders, Akbari et al. studied the role of the dysfunction of regulatory T cells (Tregs) affecting the etiology of autism spectrum disorders (ASD). As specific Tregs are known to be regulated by a group of long non-coding RNAs (lncRNAs), in this study the authors compared the expression of five lncRNAs implicated in Treg regulation in blood samples from ASD cases and controls. These lncRNAs were FOXP3 regulating long intergenic non-coding RNA (FLICR), MAF transcriptional regulator RNA (MAFTRR), NEST (IFNG-AS1), RNA component of mitochondrial RNA processing endoribonuclease (RMRP), and Th2 cytokine locus control region (TH2-LCR). The group concluded that their study supports dysregulation of Treg-related lncRNAs in patients with ASD and suggests these lncRNAs could be used as peripheral markers for ASD.

Xu et al. recently identified five-novel heterozygous *ARFGEF1* variants implicated in neurodevelopmental disorders. It is already known that mono-allelic loss-of-function variants in *ARFGEF1* caused a developmental delay, intellectual disability, and epilepsy, of varying clinical severity. The group demonstrated that variants in this gene affected males more severely than females in terms of acquiring neurodevelopmental disorders. This study broadens the genotypic spectrum of *ARFGEF1*-related neurodevelopmental disorder using data from a local Chigene database, identifying five novels heterozygous (likely) pathogenic variants. The group provide further evidence of the pathogenicity of *ARGEF1* haploinsufficiency in male patients and examine the likelihood of digenic inheritance in female patients.

Finally, Zheng et al. studied the role of a dominant genetic rare disease caused by the mutation in the *USP7* gene (^*^602519) on chromosome 16p13.2 in Hao-fountain syndrome (HAFOUS) neurodevelopmental syndrome. The group identified three variants, including one frameshift variant (c.247_250delGAGT) and two missense variants (c.992A>G, c.835T>G) that have not been previously reported. The predominant clinical manifestations in affected individuals were developmental disability/intellectual disability (DD/ID), abnormal behavior, language impairment, and abnormal brain magnetic resonance imaging (dilation of Virchow-Robin spaces, dilation of lateral ventricles, dilated third ventricle, abnormal cerebral white matter morphology in bilateral occipital lobes, arachnoid cyst, hypodysplasia of the corpus callosum, delayed myelination, widened subarachnoid space); some also had facial abnormalities, a small percentage had genitourinary abnormalities.

Taken together, this collection of papers provides a useful and interesting summary of how disruptions to a number of important developmental regulatory genes can lead to neurodevelopmental disorders.

## Author contributions

All authors listed have made a substantial, direct, and intellectual contribution to the work and approved it for publication.

## Funding

JOM's research is supported by the Simons Initiative for the Developing Brain (SFARI 529085).

## Conflict of interest

Author TCLSS was employed by Centogene GmbH. The remaining authors declare that the research was conducted in the absence of any commercial or financial relationships that could be construed as a potential conflict of interest.

## Publisher's note

All claims expressed in this article are solely those of the authors and do not necessarily represent those of their affiliated organizations, or those of the publisher, the editors and the reviewers. Any product that may be evaluated in this article, or claim that may be made by its manufacturer, is not guaranteed or endorsed by the publisher.

